# Congruence of Transcription Programs in Adult Stem Cell-Derived Jejunum Organoids and Original Tissue During Long-Term Culture

**DOI:** 10.3389/fcell.2020.00375

**Published:** 2020-07-02

**Authors:** Bart van der Hee, Ole Madsen, Jacques Vervoort, Hauke Smidt, Jerry M. Wells

**Affiliations:** ^1^Host-Microbe Interactomics Group, Department of Animal Sciences, Wageningen University & Research, Wageningen, Netherlands; ^2^Laboratory of Microbiology, Wageningen University & Research, Wageningen, Netherlands; ^3^Animal Breeding and Genomics, Department of Animal Sciences, Wageningen University & Research, Wageningen, Netherlands; ^4^Laboratory of Biochemistry, Wageningen University & Research, Wageningen, Netherlands

**Keywords:** intestinal organoids, porcine organoids, gastrointestinal, organoid stability, IPEC-J2

## Abstract

The emergence of intestinal organoids, as a stem cell-based self-renewable model system, has led to many studies on intestinal development and cell-cell signaling. However, potential issues regarding the phenotypic stability and reproducibility of the methodology during culture still needs to be addressed for different organoids. Here we investigated the transcriptomes of jejunum organoids derived from the same pig as well as batch-to-batch variation of organoids derived from different pigs over long-term passage. The set of genes expressed in organoids closely resembled that of the tissue of origin, including small intestine specific genes, for at least 17 passages. Minor differences in gene expression were observed between individual organoid cultures. In contrast, most small intestine-specific genes were not expressed in the jejunum cell line IPEC-J2, which also showed gene expression consistent with cancer phenotypes. We conclude that intestinal organoids provide a robust and stable model for translational research with clear advantages over transformed cells.

## Graphical Abstract

Porcine intestinal organoids show batch-independent homogeneity to their derived host and stability during long-term culture, as well as profound benefits over transformed cell cultures.

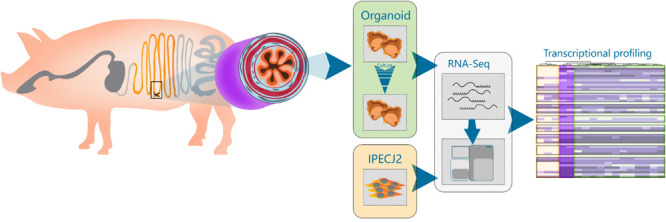

## Introduction

The intestinal epithelium plays an essential role in the digestion and absorption of nutrients while also maintaining homeostasis with symbiotic microbiota (Van Der Flier and Clevers, 2009; Wells et al., 2011; Bron et al., 2012). The physical containment of microbes to the lumen and homeostasis of tolerance and immunity depends on the functions of different lineages of intestinal epithelial cells (Van Der Flier and Clevers, 2009; Wells et al., 2011). For decades, scientists have exploited the replicative potential of immortalized intestinal cells as enterocyte models to study host-pathogen interactions and intestinal functions *in vitro*. Such monotypic cell models have led to important discoveries but have notable limitations. Immortalized cell lines can undergo significant genotypic alterations within a few passages *in vitro* which are potential threats to data reproducibility (Gillet et al., 2013; Gisselsson et al., 2018; Liu et al., 2019). Furthermore, cell lines often have altered pathway expression compared to primary cells (Gillet et al., 2013). Since 2009, when it was shown that intestinal adult leucine-rich repeat-containing G protein-coupled receptor 5 (LGR5+) stem cells (Barker et al., 2007) could be grown into organotypic cultures and propagated in 3D culture (Ootani et al., 2009; Sato et al., 2009), there has been much interest in employing intestinal organoids as advanced models. A distinct advantage of organoids is the development of a crypt-villus axis with a similar spatial organization of the heterotypic cell lineages found in the tissue of origin. Additionally, methods for generating polarized monolayers of organoids cells (Moon et al., 2014) have been optimized (van der Hee et al., 2018) to improve the versatility of the models, e.g., to study transport, and differential responses to luminal or basolateral stimulants. Another favorable property of organoids generated from adult intestinal stem cells is that they express specific functions associated with their original intestinal location (Middendorp et al., 2014). This means that location-specific functions of different parts of the intestine are intrinsically programmed in adult stem cells.

In the future we can expect intestinal organoids to be increasingly adopted as intestinal models for humans and other mammals. However, there are some unresolved issues that need to be addressed to ensure reliability and reproducibility of results in this emerging field. As organoids contain different cell types there is potential for variability and problems with reproducibility which may compromise their application to phenotype individuals. To address this issue, we assessed the transcriptional stability of intestinal organoids differentiated from the same crypt batch and between organoids from different pigs (*Sus scrofa domesticus*) over long-term passage. Furthermore, we compared expressed genes and pathways in organoids, the original epithelial tissue from which the organoids were derived, and IPEC-J2, a porcine cell line derived from the jejunum. The results show that intestinal organoids derived from adult stem cells generally resemble the epithelial tissue of origin in terms of expressed genes and provide a reference for researchers wishing to investigate specific small intestinal functions not present in IPEC-J2.

## Materials and Methods

### Intestinal Organoid Generation

Jejunum tissue segments were obtained from control piglets used for another study, following guidelines of the animal ethics committee of Wageningen University. Two 5-week-old piglets were used for generating organoids following procedures previously described (Sato et al., 2011; van der Hee et al., 2018). Briefly, a 2 cm section of the mid-jejunum was dissected and placed in ice-cold PBS. After opening the sections longitudinally, jejunum segments were washed three times in ice-cold PBS and villi removed by carefully scraping with a scalpel. Small sections of the mucosa were cut from the muscle layer, divided into small cubes, and transferred into ice-cold PBS containing 30 mM EDTA and incubated with rotation at room temperature for 15 min. The PBS – EDTA was then replaced and incubation continued for 10 min at 37°C. After washing in ice-cold DMEM supplemented with 5% penicillin/streptomycin (Gibco, Thermo Fisher Scientific), the crypts were dissociated by rigorous vortexing and passed through a 100 μm strainer into cold DMEM containing 5% fetal bovine serum (FBS, v/v). Crypts were pelleted by centrifugation at 300 × *g* for 5 min, and suspended in Matrigel (Basement Membrane, Growth factor reduced, REF 356231, Corning, Bedford, MA, United States). To improve surface tension, empty 24-well plates were pre-incubated overnight at 37°C. Seven drops of Matrigel containing crypts were placed in each well (approx. 35 μl per well) and inverted to polymerize at 37°C. After polymerization, 600 μl F12 cell culture medium (Gibco) was added, supplemented with 100 μg/ml primocin (Invivogen), 10 mM HEPES (HyClone), 1 × B-27 (Gibco), 1.25 mM *N*-acetylcysteine (Sigma), 50 ng/ml human epidermal growth factor (R&D systems), 15 nM gastrin, 10 mM nicotinamide, 10 μM p38 MAPK inhibitor (Sigma), 600 nM TGFβ receptor inhibitor A83-01, and conditioned media for recombinant Noggin (15% v/v), Spondin (15% v/v), and Wnt3A (30% v/v) provided by Dr. Kuo and Hubrecht Institute (Utrecht, Netherlands). Organoids were passaged at a 1:5 ratio every 5 days using ice-cold DMEM by mechanical dissociation, centrifugation at 500 × *g* for 5 min, and plating in fresh Matrigel matrix droplets as previously described (van der Hee et al., 2018). For measuring transport of amino acids (AAs), two-dimensional organoids were generated as previously described (van der Hee et al., 2018). 3D organoids were dissociated into single cells by TrypLE digestion and seeded on Matrigel (0.5% v/v) precoated transwells (Falcon BD). After reaching confluence, the apical medium was replaced with DMEM (Gibco) and basolateral medium with HBSS (Gibco). After incubation, the basolateral AA composition was measured using triple quadrupole mass spectrometry (TQMS).

### Culturing Methods and RNA Isolation

Directly after crypt isolation, organoid cultures were separated into three batches per animal and grown independently for 17 passages. Jejunum organoids were grown for 3 and 12 weeks (4–17 passages) and extracted using ice-cold PBS. After washing twice in PBS, intact organoids were pelleted at 300 × g for 5 min and suspended in RLT lysis buffer and stored at −80°C prior to RNA isolation. Porcine jejunum epithelial cell line IPEC-J2 (ACC-701) was grown in DMEM F12 medium supplemented with 10% FBS and 5% penicillin/streptomycin (P/S, Gibco) in 75 cm^2^ culture flasks. Data for one IPEC-J2 (p67) transcriptome was kindly provided by Dr. Richard Crooijmans, via the Functional Annotation of ANimal Genomes (FAANG, BioSamples accession SAMEA4447551) (Giuffra et al., 2018). For the analysis of the remaining two lines, IPEC-J2 at passage 87 and 91 were seeded at 5 × 10^4^ cells/well in 24-well plates and grown to confluence within 48 h with reduced P/S (1%). The monolayers were subsequently left to differentiate for 5 days, lysed using RLT-buffer, and stored at −80°C prior to RNA isolation. For RNA extraction of tissue, 0.5 mg of whole jejunum cross section per animal was added to a gentleMACS M tube (Miltenyi Biotec, Germany) and dissociated in 2 ml RLT lysis buffer using a gentleMACS Dissociator for 30 s. Subsequently, 100 μl of homogenized tissue suspension was added to 500 μl fresh RLT buffer, homogenized using pipetting with a p200 pipette, and stored at −80°C until extraction. Total RNA was extracted using a RNeasy Mini Kit (Qiagen) following manufacturer’s instructions including a 15 min on-column DNAse step. Preliminary tRNA concentrations, contamination and degradation were identified using Qubit (Thermo-Fisher) and gel-electrophoresis. The quantity and integrity of RNA was measured using a Bioanalyzer 2100 (Agilent).

### RNA-Sequencing Procedures and Data Handling

A minimum of 1 μg total RNA in 50 μl was used for library preparation using the TruSeq RNA sample preparation kit (Illumina) following the manufacturer’s protocol at Novogene. Briefly, total RNA samples were depleted for ribosomal RNA using the RiboZero kit and enriched for mRNA using oligo(dT) beads, fragmented, and synthesized into cDNA using mRNA template and hexamer primers. Custom second strand-synthesis buffer (Illumina), dNTP’s, RNAse H and DNA Polymerase I were added for second strand synthesis initiation. Furthermore, following a series of terminal repair, cDNA library construction was completed with size selection and PCR enrichment. Samples were sequenced using an Illumina Hi-Seq 4000 (Novogene, Hong Kong) at 9 GB raw data/sample with 150 bp paired-end reads.

Raw sequencing reads were checked for quality using FastQC [v0.11.5; (Andrews, 2017)] and trimmed using trim-galore for adaptors and quality [v0.4.4 (Krueger, 2017)]. Only paired-end reads longer than 35 bp were included for further downstream analysis. Sequences were aligned against Ensembl *Sus scrofa* reference genome and annotation 11.1.91 (Zerbino et al., 2018) using Tophat [v2.1.1 (Trapnell et al., 2012)]. Transcriptomes were assembled with 5 bp intron overhang tolerance, merged, normalized, and analyzed using the Cufflinks package [v2.2.1 (Trapnell et al., 2012)]. Differential expression was analyzed with 0.01 false discovery rate (FDR) using cuffdiff with bias and weight correction and visualized in R using CummeRbund [v2.7.2 (Trapnell et al., 2012)]. Mapping analytics can be found in Supplementary Table S1. Fragments per kilobase million (FPKM) were calculated and log-transformed for downstream analysis. The sequencing data was also processed using CLC Genomics Workbench 11 (Qiagen) using identical reference genome, annotation and settings for identification of insertions/deletions, breakpoints, structural variants, and track generation. Output was filtered for genes <1 FPKM to establish expression.

### Data Availability

All data are available for download through the gene expression omnibus under GEO accession number GSE146408. This data also contains the merged transcriptome file for cufflinks analysis and detailed analysis. The FPKM value data table is available as Supplementary File S1.

### Functional Analysis and Pathway Expression

Differentially expressed genes were clustered by k-means into seven clusters using CummeRbund. Due to limited analysis methods for further downstream functional analysis in pig, databases for human genomes were used as a background. Differentially expressed genes were analyzed for functional enrichment and ontologies using the TOPPfun suite (Chen et al., 2009), and gene list enrichment and candidate prioritization were evaluated with a threshold <0.05 for *P*- and *Q*-value adjusted for FDR with the Benjamini-Hochberg procedure (B&H). Ingenuity Pathway Analysis (IPA, Qiagen) was used for determining overlapping networks of expressed genes between tissue and organoids, and overall expression of molecular and cellular development of organoids between time-points. Genes for elevated tissue-specific expression were acquired from the human protein atlas for specific mRNA transcription in the small intestine, and porcine orthologs were identified to determine tissue and group enriched genes (Fagerberg et al., 2014).

### Histological Analysis

Whole mount imaging was performed as previously described with small modifications (Dow et al., 2015). Organoids grown in 8-well chambered slides (Millicell EZ slide, Merck) were fixed in 4% paraformaldehyde (PFA) for 2 h at RT. PFA-fixed organoids were stained using FITC-conjugated UEA-1 antibody (1:250, FL-1061, Vector Laboratories, United States) for 2 h and counterstained with Hoechst (0.5 μg/ml, 33342, Thermo Fisher) for 10 min at room temperature. Z-stacks of whole organoids were imaged on a confocal microscope (Zeiss). Immunohistochemical analysis for Mucin-2 was performed following procedures previously described (Loonen et al., 2014; van der Hee et al., 2018). Organoids were retrieved from Matrigel using ice-cold PBS, pelleted, and fixed overnight using 1% PFA at 4°C. After pelleting, organoids were suspended in 2% agarose gel, dehydrated and embedded in paraffin blocks. After cutting 5 μM-thick sections and subsequent drying on glass slides, sections were rehydrated, blocked in 5% normal goat serum, and stained using anti-MUC2 antibody (1:200, AB_1950958, GeneTex) overnight. After addition of secondary FITC-antibody (Thermo-Fisher), sections were counterstained using Hoechst and imaged using a DM6 microscope fitted with DFC365 camera at 40x magnification.

## Results

### Comparative Analysis of the Transcriptome of Organoids, Tissue and a Cell Line Derived From the Porcine Jejunum

**FIGURE 1:**
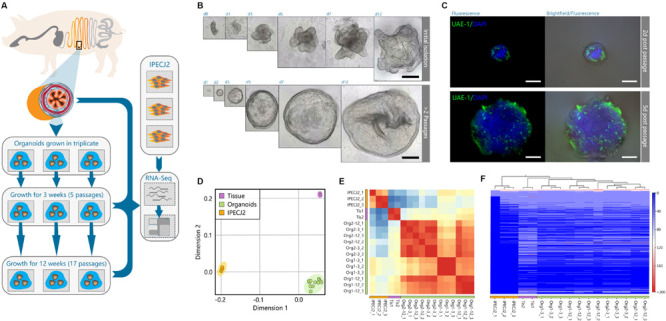
Porcine intestinal organoid culture and transcriptome sequencing overview. **(A)** graphical overview of the study design. Organoids were generated from the jejunum of two individual pigs, directly divided into triplicate organoid cultures per animal, and passaged for 12 weeks. Total RNA of tissue, organoids, and jejunum cell line IPEC-J2 was extracted and sequenced by RNA-seq. **(B)** Initially after isolation, intestinal crypts form budding organ-like structures *in vitro*. Within 2 weeks of passaging, organoids form more spheroid-resembling structures for the remainder of the experiment, indicating more long-term reproducibility after a short-term series of passaging. **(C)** After 12 weeks of growth, spheroids still retain secretory cell lineage differentiation early post-passaging (green: stained with UEA-1). **(D)** Multidimensional scaling of transcriptomic data showed separation of organoids, tissue and IPEC-J2, where organoids and tissue show separation in only one dimension. **(E)** Correlation matrix of all samples show high correlation among individual organoid batches, and strong correlation between tissue. **(F)** Heat map and hierarchical clustering of all expressed genes (>1 FPKM) in the dataset.

Jejunum tissue was isolated from two euthanized 5-week-old piglets for generating organoids and isolation of RNA from epithelial cells (Figure 1A). Triplicate batches of each organoid were separately cultured for 12 weeks by passaging approximately every 5 days. After 3- and 12-weeks continuous culture (4–17 passages) RNA was isolated from the organoids for RNA sequencing (Figure 1A). Within the first two passages after isolation, the organoids acquired a budding phenotype, which might be attributed to whole crypt isolation also containing transit amplifying cells in differentiation stages; as opposed to basal culture after 2 weeks forming a more cyst-like phenotype. After 2 weeks continuous culture the organoids formed spheroids (Figure 1B) but maintained different cell lineages, as shown with UEA-1 staining for secretory cell lineages (Figure 1C and Supplementary Figure S1). Similarly, RNA was isolated from the porcine jejunum cell line IPEC-J2 at passage number 67, 87, and 91. RNA sequencing data was analyzed using a customized analysis pipeline and CLC genomics workbench 11. Multidimensional scaling showed the transcriptomes of the organoids clustered closely together, despite 12-weeks continuous passage and isolation from different pigs (Figure 1D). It was also evident that the transcriptomes of the tissue and organoid samples were most similar, not separating in the first dimension, whereas IPEC-J2 separated from tissue in both dimensions. A correlation matrix of transcriptomic data from all samples revealed highest similarity for replicate samples of the same origin (Figure 1E).

The hierarchical clustering of gene expression (Figure 1F) further revealed that the transcriptome of organoids more closely resembled that of jejunum epithelial tissue than the IPEC-J2 cell line. The two tissue samples clustered under the same branch, where organoids showed consistent homogenous expression derived from different pigs than between 3 and 12- week cultures of replicate samples of the same organoid batch. Furthermore, organoid transcriptomes showed better correlation to tissue (*r* = 0.77) than IPEC-J2 (*r* = 0.73), whereas between tissue and IPEC-J2 correlation is lower (*r* = 0.57) (Supplementary Figure S2).

### Organoid Transcriptomes Closely Resemble Gene Expression Signatures Associated With Their Tissue of Origin

**FIGURE 2:**
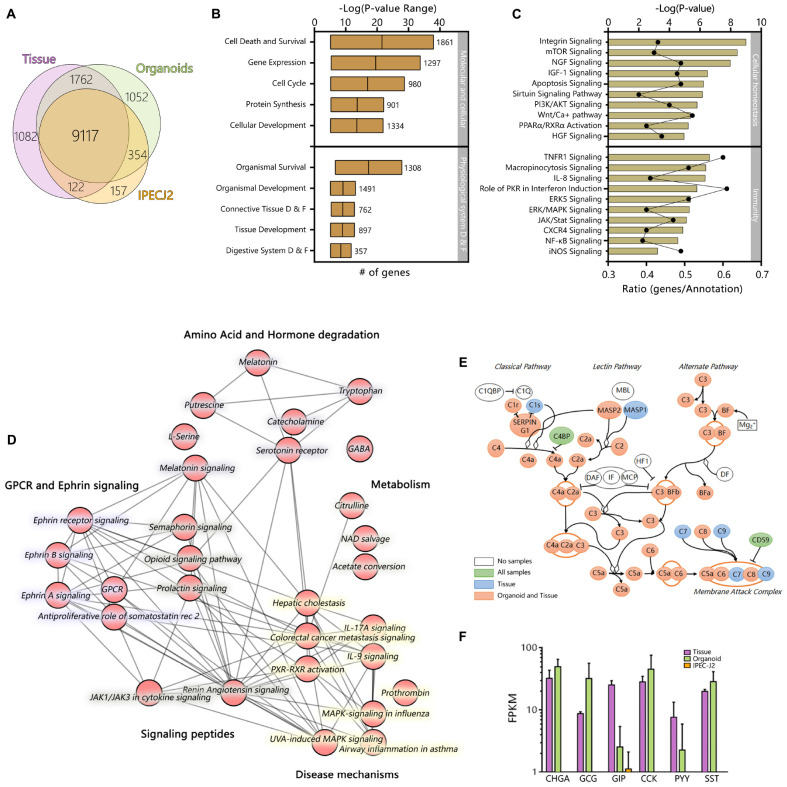
The transcriptome of jejunum organoids exhibits strong similarity to its derived tissue transcriptome, including a distinct group of overlapping genes not expressed in IPEC-J2. Averages of all expressed genes were compared between sample type and **(A)** can be viewed in the weighted Venn-diagram. All genes expressed in organoids after 12 weeks of culture were analyzed using Ingenuity pathway analysis. **(B)** Molecular, cellular, and physiological system development and function shows many genes involved in basic cellular and tissue specific processes. More than >400 pathways were expressed in the organoid RNA-seq dataset. **(C)** The top 10 cellular homeostasis and immunity related pathways; –logP values indicate statistical probability of pathway expression; ratio, indicates number of expressed genes divided by number of annotated genes in the pathway. Testing the RNA-seq dataset for overlapping genes revealed a set of 1762 genes exclusively expressed in tissue and organoids. **(D)** Top 30 connected canonical pathways of these 1762 genes from ingenuity pathway analysis, which showed subdivision into metabolic, disease, GPCR/Ephrin signaling, and small molecule degradation pathways. **(E)** Expression of genes involved in the complement pathway are expressed in organoids and tissue (Pink), Tissue only (Blue), organoids tissue and IPEC-J2 (Green), or not found to be expressed (White). **(F)** Expression patterns of genes involved in Enteroendocrine signaling [CHGA, Chromogranin A; GCG, Glucagon; GIP, Gastric inhibitory polypeptide; CCK, Cholecystokinin; PYY, Peptide YY; SST, Somatostatin; Purple, Tissue; Green, Organoid; Orange, IPEC-J2, data shown as Log(FPKM)].

Of the 24912 annotated genes in the pig genome, 11099 (44.6%) were not expressed in the dataset (<1 FPKM). All samples shared expression of 9117 genes, and a large set of genes was commonly expressed between organoids and tissue exclusively (1762 genes; Figure 2A). Genes associated with different overlapping areas of the Venn diagram were categorized by gene ontology using TOPPfun (Supplementary File S2).

However, the dataset for expressed genes in organoids still contained 1304 genes not annotated denoted with an unknown gene ID, and after conversion to human orthologs using g:profiler (Reimand et al., 2016), only 121 of these unknown ID’s with unknown function acquired a gene annotation. Nevertheless, after conversion most of the 1304 genes did contain a description (80.6%), but no gene ortholog name to identify specific function. It is therefore evident that further curation of the porcine ontology database is necessary to generate a more comprehensive reference genome for transcriptomics research.

The top clusters of all pathways, based on all expressed genes, in organoids are involved in basal molecular and cellular function as well as physiological system development, reflecting the interactions with extracellular factors and self-organization of organoid microanatomy (Figure 2B). Pathways of particular relevance for using organoid models in host-microbe interactions, such as homeostasis and innate immunity, were highly expressed. Cellular homeostatic signaling or activation pathways included integrin, mTOR, Sirtuin, PPARα, and RXRα, with typically more than 40% of genes in the annotation being expressed (Figure 2C). Immunity- pathways included important cytokine and cytokine receptor signaling pathways (e.g., *TNFR1, IL8, JAK/STAT, PKR*) and innate immune signaling (*NF-kB, ERK/MAPK, iNOS*).

Ingenuity pathway analysis of the 1762 expressed genes shared only between organoids and tissues revealed pathways associated with GPCR and Ephrin signaling, which is associated with processes such as cell migration and stem cell differentiation (Figure 2D). Pathways associated with the endocrine functions of cells were also identified including signaling via tryptophan derived melatonin and serotonin. Other pathways specific to organoids and tissue included pathways linked to cytokine, MAP-kinase and other signaling pathways, which are altered in various disease states. Genes encoding complement factors were also specifically expressed in tissue and organoids. Recent studies integrating intestinal transcriptomes deposited in public databases suggest that intestinal expression of complement pathways plays a homeostatic role, being upregulated by inflammatory challenges to control microbial invasion or colonization (Sina et al., 2018; Benis et al., 2019). From this data it is evident that many complement factors are expressed in organoids and tissue (Figure 2E). A key difference between IPEC-J2 and organoid or tissue was the specific expression of hormones associated with Enteroendocrine cells, such as *CCK* which induces secretion of digestive enzymes and *PYY*, a satiety hormone (Figure 2F).

**FIGURE 3:**
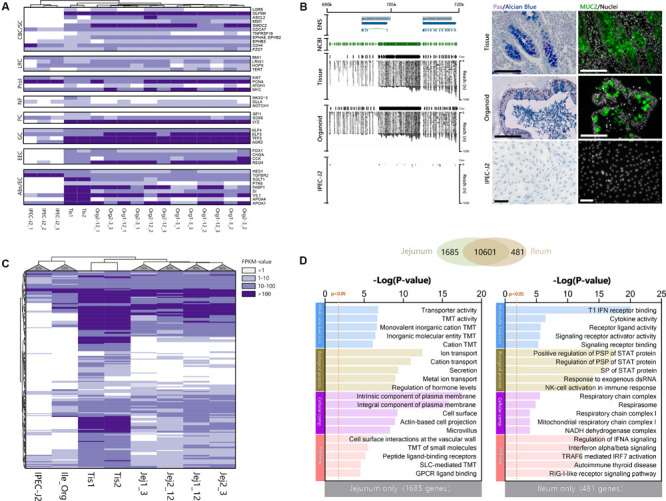
Organoids derived from adult intestinal stem cells show intrinsic programming to differentiate into different epithelial cell lineages and express small intestine-specific genes. **(A)** Cell type-specific transcripts for Crypt Base Columnar *(CBC)* and Stem cells, Label-retaining *(LRC)* +4 cells, Proliferation (Prol), Niche factors (NF), Paneth cells (PC), Goblet cells (GC), Enteroendocrine cells (EEC), and Absorptive cells or enterocytes (Abs/EC). **(B)** Overlaying the NCBI gene tracks of MUC2 NC_010444.4 on chromosome 2 at location 689363–719542bp (green area), shows identical overlap with the mapped reads and coverage (Cov) from organoid and tissue samples, but not in IPEC-J2 (ENS; Ensembl reference genome). To confirm MUC2 protein translation and subsequent mucus formation, Carnoy fixed tissue, organoid, and IPEC-J2 samples were stained with PAS/Alcian blue (left) and porcine anti-MUC2 (right; black and white size bars indicate 100 μm). **(C)** Most small intestine-specific genes identified in the human protein atlas are also expressed in porcine jejunum tissue and their derived organoids (>74%), whereas fewer are expressed in ileum organoids (52%). IPEC-J2 only expressed 32% of the small intestine-specific genes. **(D)** Organoid transcriptomes from jejunum and ileum were compared to identify differences in expressed genes, showing large overlap of genes (Venn), but also some differences. The different genes were analyzed using TOPPfun to identify putative differences in gene ontology and pathways (TMT, Transmembrane transport; PSP, Peptidyl-serine phosphorylation).

Genes which are specific for the different cell lineages found in the small intestinal epithelium (Grun et al., 2015), were generally highly expressed in organoids (Figure 3A) but largely absent in IPEC-J2. Surprisingly IPEC-J2 lacked expression of some genes commonly associated with mature absorptive enterocytes, even though cultures of this cell line are reported to differentiate into functional epithelium (Vergauwen, 2015). Initially our dataset suggested lack of mucin 2 (*MUC2*) gene expression using Ensembl gene annotation. However, we identified a high number of RNA-seq reads from organoids and tissue mapping to the chromosomal locus associated with *MUC2* in the NCBI database (XM_021082584, Chromosome 2, bp 689,363–719,542) (Figure 3B). We verified the expression of *MUC2* in porcine organoids and tissue using histology confirming mucin production and secretion as described previously (Sato et al., 2011; van der Hee et al., 2018). The genes identified in the human protein atlas to be enriched in the small intestine (i.e., jejunum) (Berglund et al., 2008; Fagerberg et al., 2014) were checked for expression in porcine jejunum tissue, jejunum and ileum organoids, and IPEC-J2 cells (Supplementary File S3). Most of the porcine orthologs (65% in tissue samples) were indeed expressed in epithelial tissue from the pig jejunum (Figure 3C). Moreover, between 74 and 86% of the porcine jejunum tissue-specific genes were also expressed in jejunum organoids, while this number was lower in ileum-derived organoids (52%). Furthermore, only 32% of small intestine-specific genes were expressed in IPEC-J2 cells. Genes associated with general digestion (*SI*, *FABP1*), absorption (solute-carriers; *SLC*-genes), or immunity (*IL22RA1*) were exclusively expressed in the tissue and jejunum organoids. Some of the genes associated with secreted peptides for immunity and digestion were not expressed in organoids and IPEC-J2 (i.e., *MEP1A*, *MEP1B*, *AQP10*, *CCL11*). It was shown that the intestinal epithelium is intrinsically programmed to differentiate into location specific organoid cultures (Middendorp et al., 2014). To investigate this for our organoid lines, we identified overlapping and exclusively expressed genes between jejunum and ileum-derived organoids. Both organoid types shared a large set of genes (10601), but several genes were identified for jejunum (1685) or ileum (481) only (Figure 3D). These genes were subsequently analyzed for gene set function using TOPPfun. Jejunum exclusively expressed genes associated with transmembrane transport, like solute-carrier transporters (SLC), and digestion (e.g., FABP1 and 2) (See Supplementary File S4 for output and gene lists). Ileum expressed genes associated with immune function, e.g., type 1 interferon responses by interferon alpha genes, as well as fucosyltransferase activity (FUT1 and FUT2).

### Cluster Analysis of Differentially Expressed Genes (DEGs)

**FIGURE 4:**
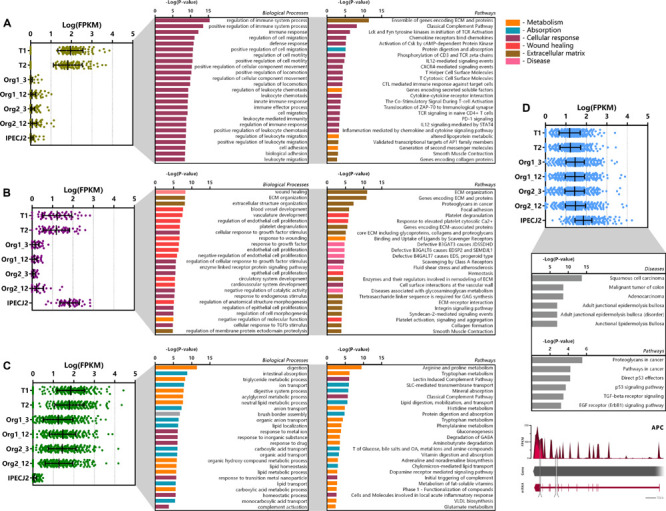
Cluster analysis of differentially expressed genes (DEGs). DEGs were clustered according to their expression pattern using k-means clustering, and subsequently analyzed for functional enrichment using TOPPfun. **(A)** Cluster of genes (*n* = 141) showing increased expression in tissue only and low expression in organoids and IPEC-J2. **(B)** Cluster of genes (*n* = 52) showing high expression in tissue and IPEC-J2, with low expression in organoids. **(C)** Cluster of genes (*n* = 299) with increased expression in tissue and organoids, with low expression in IPECJ-2 (data represented in Log(FPKM) or –Log(*P*-value), *P* and *q* < 0.05). **(D)** Cluster of genes (*n* = 841) expressed more highly in IPEC-J2 than tissue and organoids which were associated with diseases and pathways involved in tumor formation. A common mutation in colon cancer is inactivation of adenomatous polyposis coli (APC) gene where IPEC-J2 shows an insertion (217 bp, tandem duplication) in the protein coding region and splice site deletion (331 bp, cross mapped breakpoints; bottom).

Genes which were differentially expressed in tissue, organoids or IPEC-J2 (*P* < 0.05), were categorized by K means clustering and represented as biological processes and pathways (Figures 4A–C). The genes expressed only in tissue (*n* = 141) are mostly involved in immune pathways e.g., T cell and leukocyte functions, suggesting they might be due to presence of lamina propria immune cells in the tissue sample (Figure 4A). Genes related to extracellular matrix (ECM) or muscle contraction pathways were also differentially expressed in tissue compared to organoids and IPEC-J2.

The cluster of genes expressed at higher levels in tissue and IPEC-J2 (*n* = 52) compared to organoids comprise of processes related to cell morphology, proliferation, movement, remodeling of the ECM as well as integrin and ECM signaling pathways (Figure 4B). Altered expression of some of these pathways has been reported in cancer (Jorissen et al., 2009; Viana et al., 2013). The low or absent expression of the ECM genes in organoids may be due to provision of Matrigel acting as a basement membrane substrate. Furthermore, there may be components present in the intestinal ECM capable of inducing cell integrin signaling which are not present in Matrigel. Clustering genes transcribed in significantly higher amounts in IPEC-J2 show gene-list enrichment in disease processes and pathways associated with cancer (Figure 4D). Furthermore, one of our IPEC-J2 cultures showed an insertion in the protein coding region and a large deletion in the splicing site of the adenomatous polyposis coli (*APC*) gene, which is a tumor suppressor often inactivated in colon cancer (Sakai et al., 2018).

The cluster of genes with higher expression in organoids and tissue (*n* = 299) compared to IPEC-J2 involve nutrient transport and metabolic processes such as lipid digestion and transport, protein digestion and AA metabolism (Figure 4C). Examples include metabolism of tryptophan, arginine, proline, histidine, phenylalanine and transport of glucose, bile salts, fatty acids, lipids, and vitamins.

### Congruence of the Transcriptome in Individual Organoids During Passage

**FIGURE 5:**
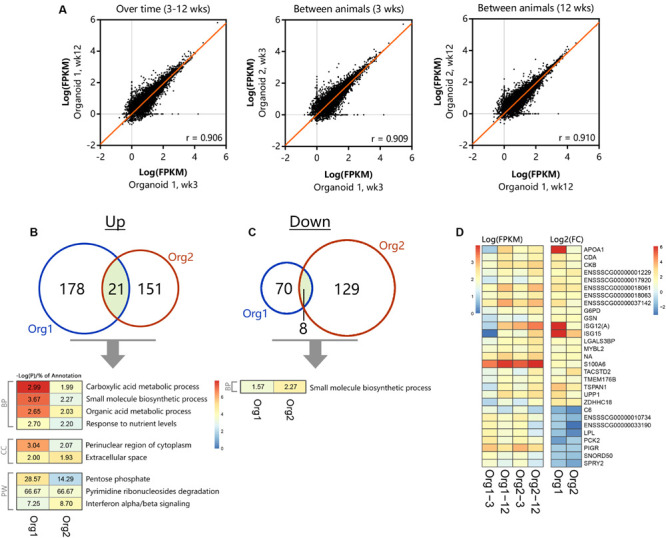
Differential gene- and pathway expression in organoids shows stable transcription over time. The two separate organoid cultures (*n* = 3 per group) were tested for expression differences between 3 and 12 weeks of culturing, **(A)** showing representative correlations over time within an organoid line and between different animals (*n* = 13813 genes with at least 1 sample >1 FPKM). Significant (*p* < 0.05) up- **(B)** and down-regulation **(C)** of genes and their corresponding overlapping pathways when tested individually using TOPPfun (overlapping area shows similar genes between organoid types, BP, Biological processes; CC, Cellular component; PW, Pathway). **(D)** Overlapping up- and down-regulated genes between 3 and 12 weeks of culturing in actual expression values [Log(FPKM)] and their corresponding fold change [Log2(FC)].

Comparison of transcriptomes of organoid cultures over time and between batches indicated high correlation in expression values (*r* = 0.906–0.910, Figure 5A). After long-term passage there were 199 genes in organoid 1 and 172 genes in organoid 2 which were significantly increased in expression (Figure 5B; *n* = 3 per group). All differentially expressed genes were distributed across nine common ontologies (Figure 5B) and consisted of only 1.93–3.67% of the annotated genes in each biological process. This included processes such as small molecule biosynthesis, organic acid metabolic processes and response to nutrient levels. Variation in expression of genes in organoid cultures over time may therefore be due to differences in nutrient abundance in culture medium, and replicative activity, rather than permanent loss or gain of functions. Long-term passage also resulted in differential down regulation of 78 and 137 genes in organoids 1 and 2, respectively (*n* = 3 per group, Figure 5C). The only common ontology found for these genes was small molecule biosynthetic process, which was also included in genes upregulated after long term passage (Figure 5B). This suggests that culture dependent conditions result in variation in a small percentage of genes in this ontology group, perhaps due to variation in number or activity of several cell types in low abundance.

The heatmap in Figure 5D shows expression or fold change in genes that were commonly differentially expressed in organoids 1 and 2 after long-term passage. Although being significantly differentially regulated, a group of genes with significantly reduced fold change in expression after 12 weeks passage was seen to be overall reduced in transcript abundance (FPKM) and appeared to be involved in unrelated processes when tested for gene ontology. Differences between organoid 1 and 2 are likely to reflect individual variation and the most striking differences were for apolipoprotein A1 (*APOA1*), *ISG12*(A), and *ISG15*.

### Small Molecule Transport by Intestinal Organoids

**FIGURE 6:**
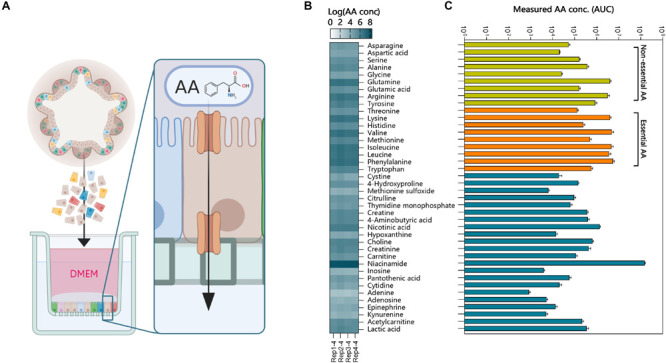
Functional amino acid transport assay using intestinal organoid monolayers. **(A)** 3-Dimensional organoids were dissociated into single cells and plated on Matrigel precoated (0.5% v/v) Transwell inserts. The monolayers were grown to confluence for 1 or 4 days and generated TEER values >750 Ω/cm2. The apical medium was replaced with DMEM and basolateral medium with HBSS. Amino acids (AA) and other molecules were measured in the basal compartment using TQMS. **(B)** AA concentrations (Log values) of individual amino acids and other molecules for 4 replicate Transwell inserts of ileum organoids. **(C)** AA concentrations in the basolateral compartment show increased transport of essential AA’s (orange).

Determining cellular function by gene expression could result in biased interpretation. For this, functional assays can be used to determine cellular function *in vitro*. The luminal compartment of 3D organoids is in the center, preventing access for apical stimulation with compounds. Methods have been developed to inject compounds into organoids (Supplementary Figure 3A), but this temporarily disrupts the barrier, and injection of multiple organoids is laborious. We demonstrated basolateral efflux of compounds by adding celltracker red dye to 3D organoids and monitoring transport into the lumen (Supplementary Figure 3B). It was evident this was an active transport process, as the dye rapidly accumulated in the center of the organoids and was as inhibited by 200 nM zosuquidar, an inhibitor of the P-glycoprotein efflux pump, involved in multi-drug resistance. To make the apical side of the organoid epithelium more accessible for transport studies we generated 2-dimensional monolayers of polarized organoid cells that still retain their differentiated features (van der Hee et al., 2018) (schematic overview; Figure 6A). This allowed us to demonstrate the AA transport function of intestinal organoids (Figure 6). The individual AA’s present in DMEM were transported across the intact monolayer in sufficient concentrations to be measured by TQMS (Figure 6B). We observed low variability in AA transport between replicates when the cell monolayers had TEER values >300 Ω/cm^2^. To ensure integrity of the monolayers we used Transwell filters with *Trans-*epithelial electrical resistance (TEER) values between 750 and 850 Ω/cm^2^ (Srinivasan et al., 2015). In addition to AA we showed that a variety of other molecules such as vitamin B5, choline, and epinephrine were transported from the apical to basolateral side. A high amount of niacinamide was measured in the basal compartment, which could be attributed to free NAD^+^ (Belenky et al., 2007). Thus, polarized monolayers of small intestinal organoids provide a model for testing several compound transport mechanisms.

## Discussion

(non-)Transformed and cancer cell lines commonly display aneuploidy and mutations affecting cellular physiology, which may further change during long-term passage. Intestinal organoids are considered an attractive alternative to cell lines, but there are no studies evaluating the variability of the transcriptome in different batches of organoids generated from the same crypts and the effect of long-term culture on genomic stability and the transcriptome.

In this study, we compared gene expression in the porcine jejunum cell line IPEC-J2, organoids derived from adult stem cells of the porcine jejunum and the intestinal tissue from which they were derived. The genes highly expressed in tissue, but not organoids or IPEC-J2, were mostly involved in immune cell, ECM or muscle contraction pathways. This is most likely due to the presence of elements of the (sub)mucosa, including lamina propria immune cells in the tissue sample. Porcine intestinal organoids possessed the different epithelial cell lineages found in tissue for at least 17 passages. An interesting observation in organoids was the high expression of *LYZ* which is often associated with Paneth cells. Until recently Paneth cells were considered to be absent in the porcine small intestine (Dekaney et al., 1997; Gonzalez et al., 2013). Paneth cells facilitate regeneration of the intestinal mucosa after metaplasia and produce high amounts of *LYZ* in presence of intestinal disease or damage (Brentnall et al., 2009; Sarvestani et al., 2018).

The set of genes expressed in organoids closely resembled that of the tissue of origin, a characteristic reported for other types of organoids (Huch et al., 2015; Matano et al., 2015). Moreover, jejunum organoids expressed the majority of gene orthologs (>74%) that are specifically expressed in the human small intestine for at least 17 passages, showing that the adult stem cells retain small intestine-associated gene expression over long-term culture (Middendorp et al., 2014; Haber et al., 2017). Interestingly, fewer small intestine-specific genes were expressed in ileum-derived organoids (52%), indicating location-specific differences. We also noticed that ileum organoids expressed the fucosyltransferase-1 (FUT1) and FUT2 genes, which were absent in our jejunum organoid transcriptome. A polymorphism in these genes has been associated with decreased susceptibility to infection with specific pathogens (Meijerink et al., 2000), further highlighting the importance of selecting the correct tissue origin when studying specific host-pathogen interactions.

Overall the batch to batch variation in organoids from the same animal was low, which may have been aided by concurrent passage and consequently differentiation state. The functions of genes which significantly altered expression between organoids from different animals or different cultures suggests they arise from differences in abundance of nutrients in culture medium or replicative activity, rather than permanent loss or gain of functions.

The main described benefits of the jejunum cell line IPEC-J2 are its non-cancerous origin and stability for more than 98 passages (Geens and Niewold, 2011; Vergauwen, 2015). However, we observed multiple indications of increased expression of genes associated with cancer or tumors in IPEC-J2 (passage 67–91). For example, these included *ANXA1* and *CALD1* (De Marchi et al., 2016), as well as insertions and deletions in the tumor suppressor *APC* (Sakai et al., 2018). We conclude that porcine jejunum organoids more closely resemble jejunum tissue than IPEC-J2 and provide a robust model for gene expression studies for at least 12 weeks of culture. As such they provide an advanced model for mechanistic studies on host-microbe interactions and intestinal physiology (Li et al., 2018). Organoids are also likely to avoid changes in glycosylation patterns seen in cancer or (non-)transformed cell models, however, timepoints extending further than 3 months could still be investigated. Motivation not to do so in this study, was the assumption that researchers would utilize the model within the tested timeframe. The RNA-seq data provides a valuable resource for researchers to assess the suitability of intestinal organoids for studying specific pathways or biological processes, as well as providing a comprehensive transcriptional map of expressed genes in IPEC-J2, formerly mainly available from microarray studies.

Previous studies have shown that organoids express proteins involved in nutrient sensing, like fructose or glucose, as well as transport by several transporters, like GLUT5, SGLT1, or PEPT1 (Zietek et al., 2015; Kishida et al., 2017). We verified the ability of 3-dimensional organoids to transport small molecules into the lumen within a short amount of time. However, studying transport over the epithelial barrier requires apical stimulation with compounds. The spatial nature of 3-dimensional organoids prevents easy access to the apical surface, but can be overcome by injection or monolayer formation. We have recently shown that apical stimulation of small intestinal organoids with various protein rich ingredients elicit specific metabolic responses, like altered lipid metabolism (Kar et al., 2020). In that study, predicted putative pathways were verified using protein-detection assays, showing the ability to measure gene-predicted responses in organoids. Transport of AAs is an essential function of the small intestine, and by measuring individual AA concentrations in a monolayer Transwell system enabled us to identify the rate at which different AAs cross the epithelial barrier. In the future, such a model could be used to identify specific transporter genes and their activity using CRISPR-Cas9 gene editing methods or transport inhibitors (Zietek et al., 2015; Schwank and Clevers, 2016).

Pig organoids can be rapidly generated from left over slaughter material without requirement for ethical approval. Thus, they have potential to be used to identify new phenotypes and investigate the role of genetic polymorphisms in susceptibility to enteric infections or other production traits. Furthermore, porcine intestinal physiology is considered to closely resemble that of humans increasing the potential for translational *in vitro* studies. Moreover, recent progress in developing robust methods for generating 2D monolayers from 3D organoid cultures facilitates apical exposure to test substances and also opens up possibilities for studying epithelial transport (Wang et al., 2017; van der Hee et al., 2018).

## Data Availability Statement

The datasets generated for this study can be found in the Gene expression omnibus GSE146408.

## Ethics Statement

The animal study was reviewed and approved by the Animal Ethics committee of Wageningen University.

## Author Contributions

JW, BH, and HS conceptualized the study. BH performed the experiments, curated, analyzed, and visualized the data. JW and HS advised on the analysis methods and design of the figures. OM set up the sequencing contact and developed the analysis pipeline. JV performed the QTMS measurements and analyzed the data. JW and BH developed the methodology and wrote the initial draft of the manuscript. JW, HS, OM, JV, and BH reviewed and edited the final manuscript. All authors contributed to the article and approved the submitted version.

## Conflict of Interest

The authors declare that the research was conducted in the absence of any commercial or financial relationships that could be construed as a potential conflict of interest.
