# Supplementary material for: Congruence of Transcription Programs in Adult Stem Cell-Derived Jejunum Organoids and Original Tissue During Long-Term Culture
Source: Front Cell Dev Biol. 2020 Jul 2;8:375. doi: 10.3389/fcell.2020.00375 (PMC7343960; doi:10.3389/fcell.2020.00375)
Supplement: Supplementary file 5 [file Data_Sheet_1.docx]

**
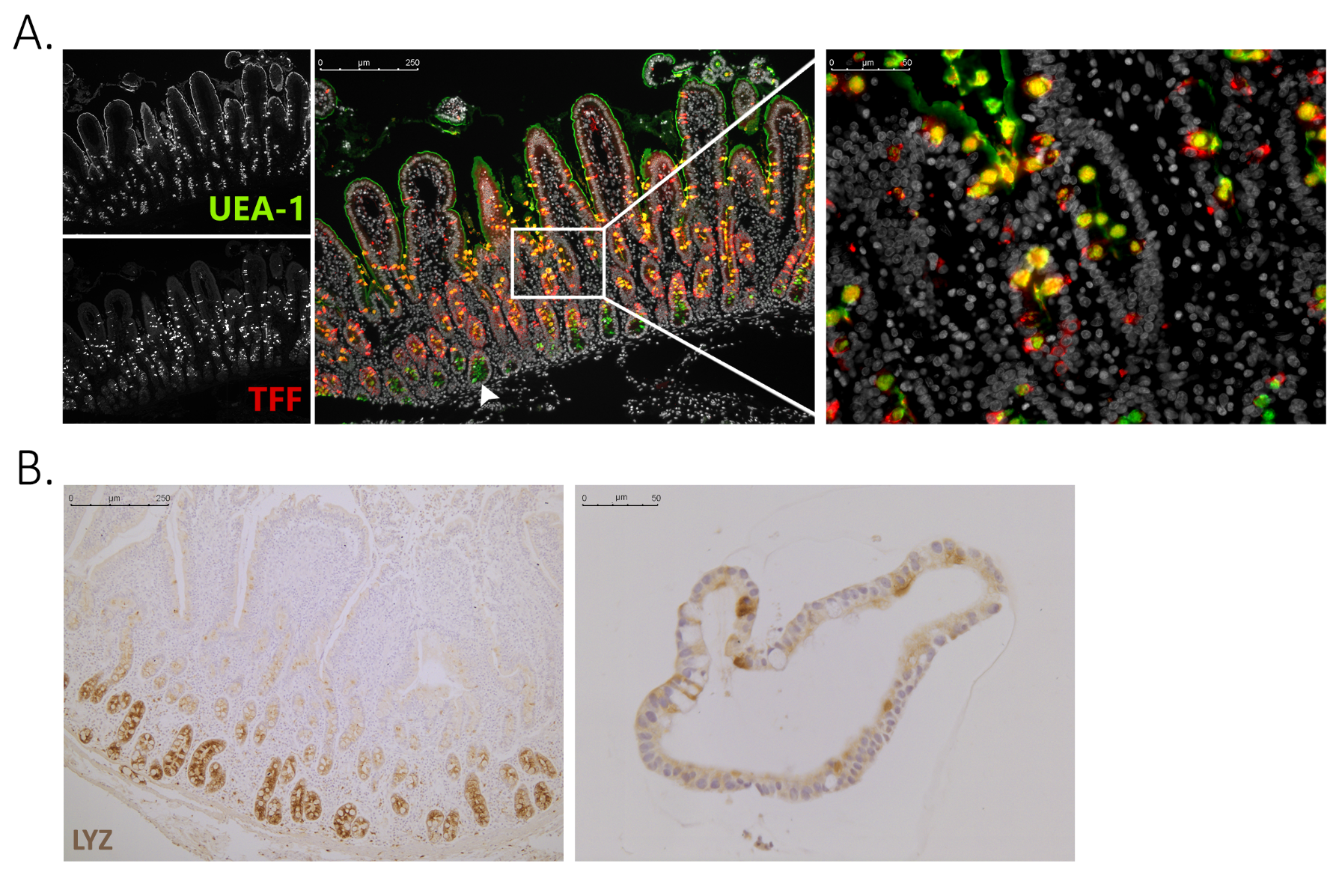
Supplementary file 5. Extended Results**

**Supplementary Figure 1. Tissue and organoid staining for secretory lineages.** (A) Double staining with UEA-1 (secretory cell lineage) and trefoil factor 3 (TFF3; goblet cell specific) shows overlapping staining, as well as staining deeper in the crypt. This indicates that UEA-1 stains for secretory cell lineages in the porcine small intestine (left: individual channels, middle: overlay, yellow is staining overlap, right: zoom panel of middle image. (B) Paneth cells have been identified in the porcine intestinal crypt by lysozyme staining (left), which recapitulates in their derived organoids (right).


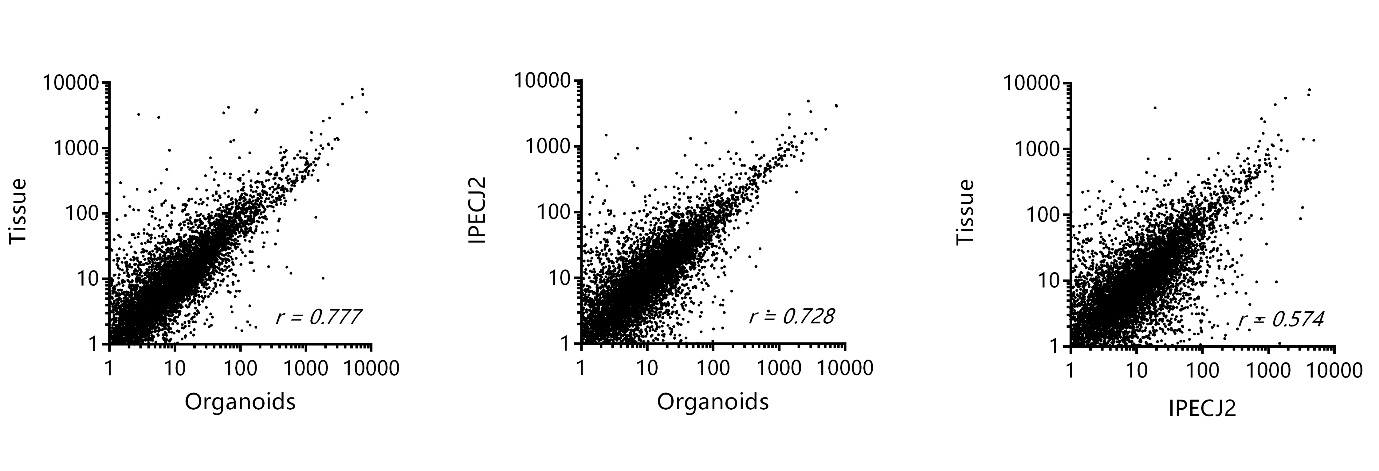


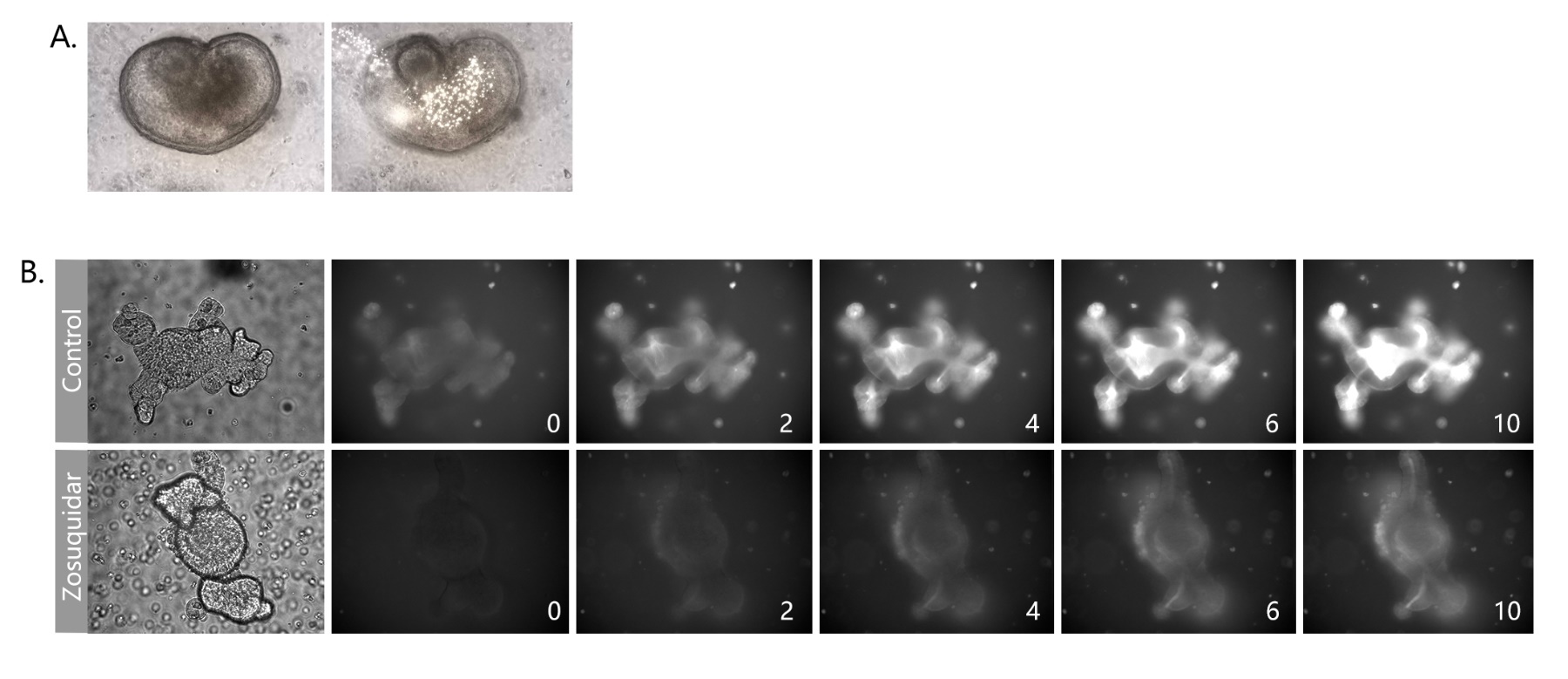
**Supplementary Figure 2. Correlation plot of average FPKM per sample type.** Organoids and tissue show strong correlation (r = 0.777), as well as between Organoids and IPEC-J2 (r = 0.728). The least amount of correlation was observed between Tissue and IPEC-J2 (r = 0.574)

**Supplementary Figure 3. Exposure of small compounds to intestinal organoids to study epithelial transport.** (A) 3-dimensional organoids can be injected with compounds and substances such as GFP+ microbeads, to stimulate the luminal compartment. (B) Small compounds can be actively transported into the intestinal organoid lumen by addition of small compounds like celltracker red (CTR; control) and inhibited by using zosuquidar (200 nM) (time in minutes).

**Supplementary Table 1. Summary of mapping RNA-seq data to reference genome Sus Scrofa 11.1.**

|  | Mapped reads | | |  |  |  |
| --- | --- | --- | --- | --- | --- | --- |
| **Sample** | ***Left*** | ***Right*** | ***Overall mapping  rate (%)*** | ***Pairs*** | ***Multiple  alignments (%)*** | ***Concordant pair  alignment (%)*** |
| Org1-3_1 | 2850198 | 2840118 | 98.7 | 2823464 | 2.2 | 98.7 |
| Org1-3_2 | 2859471 | 2847581 | 98.6 | 2830605 | 2.2 | 97.6 |
| Org1-3_3 | 2952697 | 2942165 | 98.7 | 2923881 | 2.1 | 97.7 |
| Org1-12_1 | 3031397 | 3002603 | 97.4 | 2979507 | 2.4 | 96.0 |
| Org1-12_2 | 2983917 | 2882268 | 98.5 | 2863455 | 2.5 | 97.4 |
| Org1-12_3 | 2722083 | 2711853 | 98.5 | 2693562 | 2.6 | 97.3 |
| Tissue 1 | 3030093 | 3019758 | 98.4 | 2998320 | 3.6 | 97.3 |
| Org2-3_1 | 2728229 | 2717750 | 98.6 | 2700557 | 2.6 | 97.5 |
| Org2-3_2 | 2705521 | 2696828 | 98.6 | 2679085 | 2.6 | 97.5 |
| Org2-3_3 | 2697949 | 2732830 | 98.6 | 2671617 | 2.5 | 97.5 |
| Org2-12_1 | 2228232 | 2222736 | 95.4 | 2208029 | 2.3 | 94.4 |
| Org2-12_2 | 2252779 | 2245831 | 95.8 | 2231084 | 2.4 | 94.8 |
| Org2-12_3 | 2359295 | 2333615 | 92.9 | 2315833 | 2.6 | 91.4 |
| Tissue 2 | 2558842 | 2550033 | 98.4 | 2531447 | 3.3 | 97.2 |
| IPEC-J2_1 | 3341302 | 3322134 | 98.7 | 3304568 | 1.8 | 96.7 |
| IPEC-J2_2 | 3233204 | 3258153 | 98.1 | 3210559 | 2.0 | 96.8 |
| IPEC-J2_3 | 2882496 | 2990369 | 94.8 | 2834108 | 2.1 | 91.3 |
